# Quinolones-Induced Musculoskeletal, Neurological, and Psychiatric ADRs: A Pharmacovigilance Study Based on Data From the Italian Spontaneous Reporting System

**DOI:** 10.3389/fphar.2020.00428

**Published:** 2020-04-15

**Authors:** Cristina Scavone, Annamaria Mascolo, Rosanna Ruggiero, Liberata Sportiello, Concetta Rafaniello, Liberato Berrino, Annalisa Capuano

**Affiliations:** ^1^ Campania Regional Centre for Pharmacovigilance and Pharmacoepidemiology, Naples, Italy; ^2^ Department of Experimental Medicine, Section of Pharmacology “L. Donatelli”, University of Campania “Luigi Vanvitelli”, Naples, Italy

**Keywords:** quinolones, safety, pharmacovigilance, spontaneous reporting system, musculoskeletal, neurological and psychiatric events, adverse drug reaction, Italy

## Abstract

**Background:**

The use of quinolones has been associated with the development of serious and persistent adverse drug reaction (ADR) mainly affecting muscles, joints and the nervous system. This risk has led the European Medicines Agency (EMA) to endorse some restrictions on the use of this class of antibiotic. Therefore, we performed a study to primary estimate the reporting probability of musculoskeletal, neurological, and psychiatric ADRs among quinolone generations using national data.

**Methods:**

We retrieved Individual Case Safety Reports (ICSRs) with a quinolone as suspected drug among those reported through the Campania spontaneous reporting system from January 1^st^, 2001 to April 30^th^ 2019. Moreover, we retrieved national aggregated safety data from the online public report system (RAM system) for the period from January 1^st^, 2002 to March 31^st^, 2019. Risk factors were classified as “age greater than 60 years,” “therapeutic indication,” “renal failure,” “organ transplantation,” “use of corticosteroid,” and “history of side effects”. Reporting odds ratio (ROR) was computed to evaluate the reporting probability of musculoskeletal, neurological, or psychiatric events among quinolones generations.

**Results:**

A total of 87 ICSRs with a quinolone as suspected drug that reported at least one musculoskeletal, neurological, and psychiatric adverse event were identified in the Campania spontaneous reporting system. Forty-nine (56.3%) ICSRs reported risk factors (total risk factors 59). The most reported risk factor was “age greater than 60 years” (69.5%), followed by “therapeutic indication” (16.9%), “renal failure” (5.1%), “organ transplantation” (3.4%), “use of corticosteroid” (3.4%), and “history of side effects” (1.7%). Second-generation quinolones were associated with a lower reporting probability of musculoskeletal (ROR 0.70; 95% CI 0.63–0.79), neurological (ROR 0.81; 95% CI 0.73–0.90), and psychiatric (ROR 0.55; 95% CI 0.44–0.63) ADRs compared to the third generation of quinolones.

**Conclusions:**

Our findings showed that third-generation quinolones were always associated with a higher reporting probability of musculoskeletal, neurological, and psychiatric ADRs compared to the second generation ones. Moreover, we described risk factors in more than half of our cases suggesting that the inappropriate use of quinolones is a phenomenon that may frequently predispose patients to the occurrence of these ADRs.

## Introduction

Quinolones represent a large group of broad-spectrum bactericides. These antimicrobial agents are characterized by the presence of a bicyclic core structure related to the compound 4-quinolone; those containing a fluorine atom are defined as fluoroquinolones (Walker and Wright, 1991). Quinolones inhibit DNA synthesis through the inhibition of the bacterial DNA gyrase or topoisomerase IV (Oliphant and Green, 2002). Based on their antimicrobial activity, they are classified into four generations: the first-generation agents (cinoxacin, nalidixic acid, pipemidic acid) which show moderate gram-negative activity; the second-generation quinolones (ciprofloxacin, enoxacin, lomefloxacin, norfloxacin, ofloxacin, rufloxacin), that have expanded gram-negative activity; the third-generation quinolones (gatifloxacin, grepafloxacin, levofloxacin, moxifloxacin, pefloxacin, sparfloxacin, temafloxacin) which show extended gram-negative and improved gram-positive coverage; the fourth-generation quinolones (clinafloxacin, gemifloxacin, prulifloxacin, trovafloxacin) which have expanded activity against gram-positive, gram-negative, and anaerobic bacteria (Oliphant and Green, 2002). Considering their spectrum of activity as well as their pharmacokinetic properties (they show good oral absorption and tissue penetration) they are highly effective in the treatment of several infections. Whilst the introduction of a fluorine atom on the basic quinolone structure has provided expanded activity against gram-positive bacteria and enhanced the tissue penetration of those medicines (Wright, 2000; Van Bambeke and Tulkens, 2009), it is also responsible for the occurrence of serious adverse drug reactions (ADRs) (Mehlhorn and Brown, 2007). Regarding the safety profile, data from premarketing clinical trials showed that quinolones are generally well tolerated and commonly related to the occurrence of gastrointestinal ADRs, mainly represented by nausea, vomiting, diarrhea, and constipation. Less frequently, these antibiotics can induce the occurrence of the central nervous system and dermatologic ADRs, blood toxicities, renal disorders, and skin hypersensitivity. All these ADRs seem to be generally mild and reversible (Bertino and Fish, 2000; Mandell and Tillotson, 2002). Data from postmarketing experience highlighted the occurrence of severe and life-threatening ADRs, including anaphylaxis, cardiotoxicity (QT prolongation and/or torsade de pointes), tendon rupture (this ADR rarely occurs with ciprofloxacin and levofloxacin), central nervous system ADRs (especially when quinolones are administered with anti-inflammatory drugs), dysglycemia (observed in patients treated with gatifloxacin and clinafloxacin), and hepatotoxicity (found in trovafloxacin treatment) (Kocsis et al., 2016). Due to these severe ADRs, several quinolones have been withdrawn from the market, such as temafloxacin and grepafloxacin, or their use was restricted only to the treatment of serious infections, such as the case of trovafloxacin (Mandell and Tillotson, 2002).

Recently, the safety profile of quinolones has again become a matter of public concern. Indeed, following the public hearing on those antibiotics of June 2018, the European Medicines Agency (EMA) finalized on November 2018 a review on the risk of developing serious and persistent ADRs mainly affecting muscles, joints, and the nervous system. The EMA concluded that the marketing authorization of medicines containing cinoxacin, flumequine, nalidixic acid, and pipemidic acid has to be suspended, while the use of the remaining quinolone antibiotics has to be restricted. According to this new restrictions, quinolones should not be used anymore for the treatment of infections that might get better without treatment or are not severe; nonbacterial infections; the prevention of traveler's diarrhea or recurring lower urinary tract infections; mild or moderate bacterial infections unless other antibacterial medicines commonly recommended for these infections cannot be used. Lastly, several recommendations were addressed to healthcare providers to highlight common risk factors for musculoskeletal, neurological, and psychiatric ADRs among quinolone users (Quinolone- and fluoroquinolone-containing medicinal products | EMA). Considering this recent recommendation endorsed by the EMA and the widespread use of quinolones, we decided to carry out a study using data from the Italian spontaneous reporting system to evaluate cases of musculoskeletal, neurological, and psychiatric ADRs that reported a quinolone as a suspected drug. The primary aim of our study was to estimate the reporting probability of musculoskeletal, neurological, and psychiatric ADRs among quinolone generations using national data. We also aimed to describe regional Individual Case Safety Reports (ICSRs) reporting the aforementioned drug-event couple and to describe risk factors among those ICSRs.

## Methods

### Data Sources

In Italy, the spontaneous reporting system is coordinated by the Italian Medicines Agency (AIFA) that established in 2001 the Italian spontaneous reporting database (Rete Nazionale di Farmacovigilanza – RNF) (Sessa et al., 2015). We retrieved ICSRs with at least one quinolone as suspected drug among those reported in the RNF for one Italian Region (Campania Region; South of Italy). ICSRs retrieved from the Campania spontaneous reporting system from January 1^st^, 2001 to April 30^th^, 2019 were used for the case by case evaluation and for the description of risk factors. Moreover, because in Italy is available the online public report system of ADRs (RAM system), nationally aggregated data on ICSRs and ADRs were retrieved from the RAM system from January 1^st^, 2002 to March 31^st^, 2019.

### Suspected Drugs

According to the review performed by the EMA (Quinolone- and fluoroquinolone-containing medicinal products | EMA), quinolones considered as suspected drugs were: first-generation quinolones (nalidixic acid, pipemidic acid, cinoxacin, flumequine); second-generation quinolones (enoxacin, lomefloxacin, ciprofloxacin, norfloxacin, ofloxacin, rufloxacin); third-generation quinolones (levofloxacin, moxifloxacin, pefloxacin); fourth-generation quinolones (prulifloxacin).

### ICSRs Recorded Across All the Italian Territory (RAM System)

A descriptive analysis was performed for age groups (< 1 month, 1–23 months, 2–11 years, 12–17 years, 18–65 years, > 66 years), gender, suspected drugs, and the number of ADRs, which were also stratified in those belonging to the System Organ Class (SOC) “Nervous system disorders,” “Musculoskeletal and connective tissue disorders,” “Psychiatric disorders,” or “other SOCs.”

Reporting odds ratio (ROR), its 95% Confidence Interval (95% CI) and the Chi-square test were computed to evaluate if there was a lower/higher reporting probability of musculoskeletal, neurological, or psychiatric ADRs among quinolones generations. The second-generation and the third-generation quinolones were individually used as reference groups because of the higher number of reported ADRs. A 5% significance level was considered for analysis. We computed ROR according to the current European guideline on statistical signal detection in spontaneous reporting system (European Medicine Agency, 2016).

### ICSRs Recorded in the Campania Region

Information on gender, median age, the seriousness of ADR, outcome, suspected quinolone, type of reporter, causality assessment, and the number of reported suspected drugs were provided for all ICSRs and separately for ICSRs with musculoskeletal, neurological, and psychiatric cases. Seriousness of ADRs was codified as in accordance with the International Council on Harmonization E2D guidelines (ICH E2D postapproval safety data management | EMA), whereas the outcome was categorized into six categories (recovered, improvement, resolution with sequelae, unchanged clinical condition, death, and not available) based on the national law. In order to establish the strength of the relationship between the suspected drug and the ADRs, the Naranjo algorithm (Naranjo et al., 1981) was used. All scores that ranged between possible and certain were considered reasonable for causality. All reported preferred terms (PTs) related to musculoskeletal, neurological, and psychiatric ADRs were tabled according to the SOC “Nervous system disorders,” “Musculoskeletal and connective tissue disorders,” and “Psychiatric disorders.” The time to event was computed as the difference in days between the onset date of the adverse event and the start date of quinolone therapy. A boxplot of time to events was generated for each SOC undervaluation. ICSRs reporting more than one ADR belonging to two distinct SOCs were classified as “Multiple SOC.”

In order to detect risk factors for musculoskeletal, neurological, and psychiatric cases, ICSRs were individually screened. According to the recommendation made by the EMA (Quinolone- and fluoroquinolone-containing medicinal products | EMA), the following risk factors were considered: patients aged over 60 years; renal failure; organ transplantation; use of corticosteroids; history of previous side effects to quinolones; use of quinolones for a not severe infection.

### Compliance With Ethical Standards

Safety data deriving from the Italian spontaneous reporting system are anonymous and in compliance with the ethical standard. Therefore, no further ethical measures were required (Sportiello et al., 2016; Sessa et al., 2017).

## Results

### Cases of Musculoskeletal, Neurological, and Psychiatric Adverse Events Involving Quinolones Recognized in the Italian Spontaneous Reporting System

In the period from January 2002 to March 2019, 12,059 ICSRs sent to the Italian spontaneous reporting system reported a quinolone as a suspected drug. Specifically, 51% of ICSRs reported ADRs occurred in female patients, while 41% reported ADRs occurred in male patients; in 8% of ICSRs information on gender was not reported. With regard to the distribution by age groups, most ICSRs reported ADRs occurred in the age groups 18–65 years (47%) and > 66 years (43%); in 8.3% of ICSRs age was not reported. The remaining 1.7% of ICSRs referred to ADRs occurred in the other age groups. The most-reported quinolone was levofloxacin (5,941; 49.3%), followed by ciprofloxacin (4,498; 37.3%), moxifloxacin (820; 6.8%), prulifloxacin (245; 2.0%), norfloxacin (146; 1.2%), lomefloxacin (138; 1.1%), ofloxacin (81; 0.7%), cinoxacin (70; 0.6%), pefloxacin (51; 0.4%), pipemidic acid (47; 0.4%), rufloxacin (19; 0.2%), and nalidixic acid (3; 0.0%). Because in each ICSR more than one ADR could be reported, we observed a total of 21,941 ADRs (1.8 suspected ADRs per ICSR), of which 4,418 (20.1%) were related to the SOCs “Nervous system disorders,” “Musculoskeletal and connective tissue disorders,” or “Psychiatric disorders.” Specifically, 1,681 (38.0%) ADRs belong to the SOC “Musculoskeletal and connective tissue disorders,” 1,619 (36.6%) to the SOC “Nervous system disorders,” and 1,118 (25.3%) to the SOC “Psychiatric disorders.” Distribution of the number of ADRs for quinolone reported as suspected drug and for SOC is described in **Table 1**.

**Table 1:** Number of adverse drug reactions (ADRs) distributed for the suspected drug (quinolone) and for the System Organ Class (SOC) among those reported in the Italian spontaneous reporting system.

System Organ Class					
	Nervous system disorders	Musculoskeletal and connective tissue disorders	Psychiatric disorders	Other SOCs	Total SOCs
**1° Generation**	50	19	23	353	445
• Cinoxacin	13	1	1	111	126
• Pipemidic acid	2	0	0	75	77
• Norfloxacin	35	18	22	167	242
**2° Generation**	534	514	290	6,950	8,288
• Ciprofloxacin	495	491	265	6,687	7,938
• Lomefloxacin	21	13	9	218	261
• Ofloxacin	9	7	2	125	143
• Rufloxacin	9	3	14	20	46
**3° Generation**	999	1,094	788	9,877	12,758
• Levofloxacin	796	1,025	682	8,463	10,966
• Moxifloxacin	197	52	100	1,355	1,704
• Pefloxacin	6	17	6	59	88
**4° Generation**	36	54	17	343	450
• Prulifloxacin	36	54	17	343	450
**Total**	1,619	1,681	1,118	17,523	21,941

#### Comparison of the Reporting Probability of Musculoskeletal, Neurological, or Psychiatric Adverse Events Among Quinolone Generations With the Second-Generation Quinolones as Control Group

Second-generation quinolones were associated with a lower reporting probability of musculoskeletal ADRs if compared to the third generation of quinolones (ROR 1.42, 95% CI 1.27–1.58; reference group: second-generation quinolones), and the fourth generation of quinolones (ROR 2.06, 95% CI 1.53–2.78; reference group: second-generation quinolones). No statistically significant difference in the ROR was observed with the first-generation quinolones.

For neurological ADRs, second-generation quinolones were associated with a lower reporting probability than first-generation quinolones (ROR 1.83, 95% CI 1.35–2.50; reference group: second-generation quinolones), and third-generation quinolones (ROR 1.23, 95% CI 1.11–1.38; reference group: second-generation quinolones). No statistically significant difference in the ROR was observed with the fourth-generation quinolones.

For psychiatric ADRs, the higher reporting probability was observed with the third-generation quinolones if compared to the second-generation quinolones (ROR 1.81, 95% CI 1.58–2.08). All the computations for RORs with the second-generation quinolones as reference group are reported in **Table 2**.

**Table 2:** Reporting Odds Ratio (ROR) of musculoskeletal, neurological, or psychiatric adverse drug reactions (ADRs) with quinolone generations.

**Quinolone generations**	**Control group**	**Reporting Odds Ratio of musculoskeletal ADRs** **(95% Confidence Interval)**	**P-value**
1 generation	2 generation	0.67 (0.42–1.08)	0.097
3 generation	2 generation	1.42 (1.27–1.58)	<0.001
4 generation	2 generation	2.06 (1.53–2.78)	<0.001
1 generation	3 generation	0.47 (0.30–0.76)	0.001
2 generation	3 generation	0.70 (0.63–0.79)	<0.001
4 generation	3 generation	1.45 (1.09–1.94)	0.011
**Quinolone generations**	**Control group**	**Reporting Odds Ratio of neurological ADRs** **(95% Confidence Interval)**	**P-value**
1 generation	2 generation	1.83 (1.35–2.50)	<0.001
3 generation	2 generation	1.23 (1.11–1.38)	<0.001
4 generation	2 generation	1.26 (0.89–1.79)	0.1927
1 generation	3 generation	1.49 (1.10–2.01)	0.009
2 generation	3 generation	0.81 (0.73–0.90)	<0.001
4 generation	3 generation	1.02 (0.72–1.45)	0.8953
**Quinolone generations**	**Control group**	**Reporting Odds Ratio of psychiatric ADRs** **(95% Confidence Interval)**	**P-value**
1 generation	2 generation	1.50 (0.97–2.32)	0.065
3 generation	2 generation	1.81 (1.58–2.08)	<0.001
4 generation	2 generation	1.08 (0.66–1.78)	0.7544
1 generation	3 generation	0.83 (0.54–1.27)	0.3840
2 generation	3 generation	0.55 (0.44–0.63)	<0.001
4 generation	3 generation	0.60 (0.36–0.97)	0.036

#### Comparison of the Reporting Probability of Musculoskeletal, Neurological, or Psychiatric Adverse Events Among Quinolone Generations With the Third-Generation Quinolones as Control Group

Third-generation quinolones were associated with a higher reporting probability of musculoskeletal ADRs if compared to the first generation of quinolones (ROR 0.47, 95% CI 0.30–0.76; reference group: third-generation quinolones), whereas a lower reporting probability was observed in the comparison with the fourth generation of quinolones (ROR 1.45, 95% CI 1.09–1.94; reference group: third-generation quinolones).

For neurological ADRs, first-generation quinolones were associated with a higher reporting probability than third-generation quinolones (ROR 1.49, 95% CI 1.10–2.01; reference group: third-generation quinolones). No statistically significant difference in the ROR was observed for the fourth-generation quinolones.

For psychiatric ADRs, third-generation quinolones were associated with a higher reporting probability if compared to the fourth-generation quinolones (ROR 0.60, 95% CI 0.36–0.97; reference group: third-generation quinolones). No statistically significant difference in the ROR was observed for the first-generation quinolones.

Moreover, second-generation quinolones were associated with a lower reporting probability of musculoskeletal (ROR 0.70; 95% CI 0.63–0.79), neurological (ROR 0.81; 95% CI 0.73–0.90), and psychiatric (ROR 0.55; 95% CI 0.44–0.63) ADRs compared to third-generation quinolones.

All the computations for RORs with the third-generation quinolones as reference group are reported in **Table 2**.

### Cases Recognized in the Campania Spontaneous Reporting System and Involving Quinolones

In the period from January 2001 to April 2019, 40,957 ICSRs were sent to Campania Pharmacovigilance Regional Centre, of which 442 reported at least one quinolone as a suspected drug. The median age of patients was 55 years (interquartile range, IQR: 36 - 70), with 58.6% of cases occurred in female patients. Most ICSRs were reported as not serious (289; 65.4%). Among ICSRs classified as serious, most cases were related to hospitalizations (14.9%), clinically significant conditions (12.9%), life-threatening conditions (2.0%), significant or permanent disability (1.8%), and death (0.5%). Most ICSRs reported a positive outcome classified as recovered (39.8%) or improvement (39.6%). The third- and second-generation quinolones were the most common reported suspected drugs (51.1% and 44.1%, respectively). Among quinolones, the most reported were levofloxacin (46.6%) and ciprofloxacin (40.5%). No ICSR with flumequine or nalidixic acid was found in the Campania spontaneous reporting system. The main reporter was the physician with 242 (54.8%) out of 442 ICSRs. The causality assessment was possible for 261 ICSRs (59.0%) and probable for 180 ICSRs (40.7%). Few ICSRs (14.9%) reported more than one medical product as a suspected drug. The characteristics of cases were presented in **Table 3**.

**Table 3:** Demographic characteristics and distribution for the type of reporter, suspected drug, seriousness, outcome, and causality assessment involving quinolones recognized in the Campania spontaneous reporting system from January 2001 to April 2019.

Variable	Level	Cases of musculoskeletal or neurological or psychiatric disorders (N=87)	Total (N=442)
Gender	Female Male Not available	44 (50.6) 43 (49.4) -	259 (58.6) 179 (40.5) 4 (0.9)
Age	Median (IQR)	57 (41–74)	55 (36–70)
Seriousness	Serious–other clinically significant condition Serious–death Serious–significant or permanent disability Serious–hospitalization Serious–life threatening Not defined Not serious	14 (16.1) 1 (1.1) 6 (6.7) 14 (16.1) 1 (1.1) 3 (3.4) 48 (55.2)	57 (12.9) 2 (0.5) 8 (1.8) 66 (14.9) 9 (2.0) 11 (2.5) 289 (65.4)
Outcome	Death Improvement Unchanged clinical condition Not available Recovered Resolution with sequelae	1 (1.1) 25 (28.7) 14 (16.1) 15 (17.2) 31 (35.6) 1 (1.1)	3 (0.7) 175 (39.6) 25 (5.7) 52 (11.8) 176 (39.8) 11 (2.5)
1° Generation 2° Generation 3° Generation 4° Generation	Cinoxacin Pipemidic acid Norfloxacin Ciprofloxacin Lomefloxacin Ofloxacin Rufloxacin Levofloxacin Moxifloxacin Pefloxacin Prulifloxacin	2 (2.3) 2 (2.3) - - 34 (39.1) 28 (32.2) 3 (3.4) 2 (2.3) 1 (1.1) 46 (52.9) 41 (47.1) 5 (5.7) - 5 (5.7) 5 (5.7)	10 (2.3) 4 (0.9) 4 (0.9) 2 (0.5) 195 (44.1) 179 (40.5) 12 (2.7) 3 (0.7) 1 (0.2) 226 (51.1) 206 (46.6) 18 (4.1) 2 (0.5) 11 (2.5) 11 (2.5)
Reporter	Physicians Pharmacist Other health care professional Patient/Citizen or other nonhealthcare professional figure	44 (50.6) 15 (17.2) 11 (12.6) 17 (19.5)	242 (54.8) 78 (17.6) 76 (17.2) 46 (10.4)
Causality	Possible Probable Doubt	58 (66.7) 28 (32.2) 1 (1.1)	261 (59.0) 180 (40.7) 1 (0.2)
Number of reported suspected drugs	>1 1	10 (11.5) 77 (88.5)	66 (14.9) 376 (85.1)

#### Cases Involving Musculoskeletal, Neurological, and Psychiatric Adverse Events

**Figure 1:**
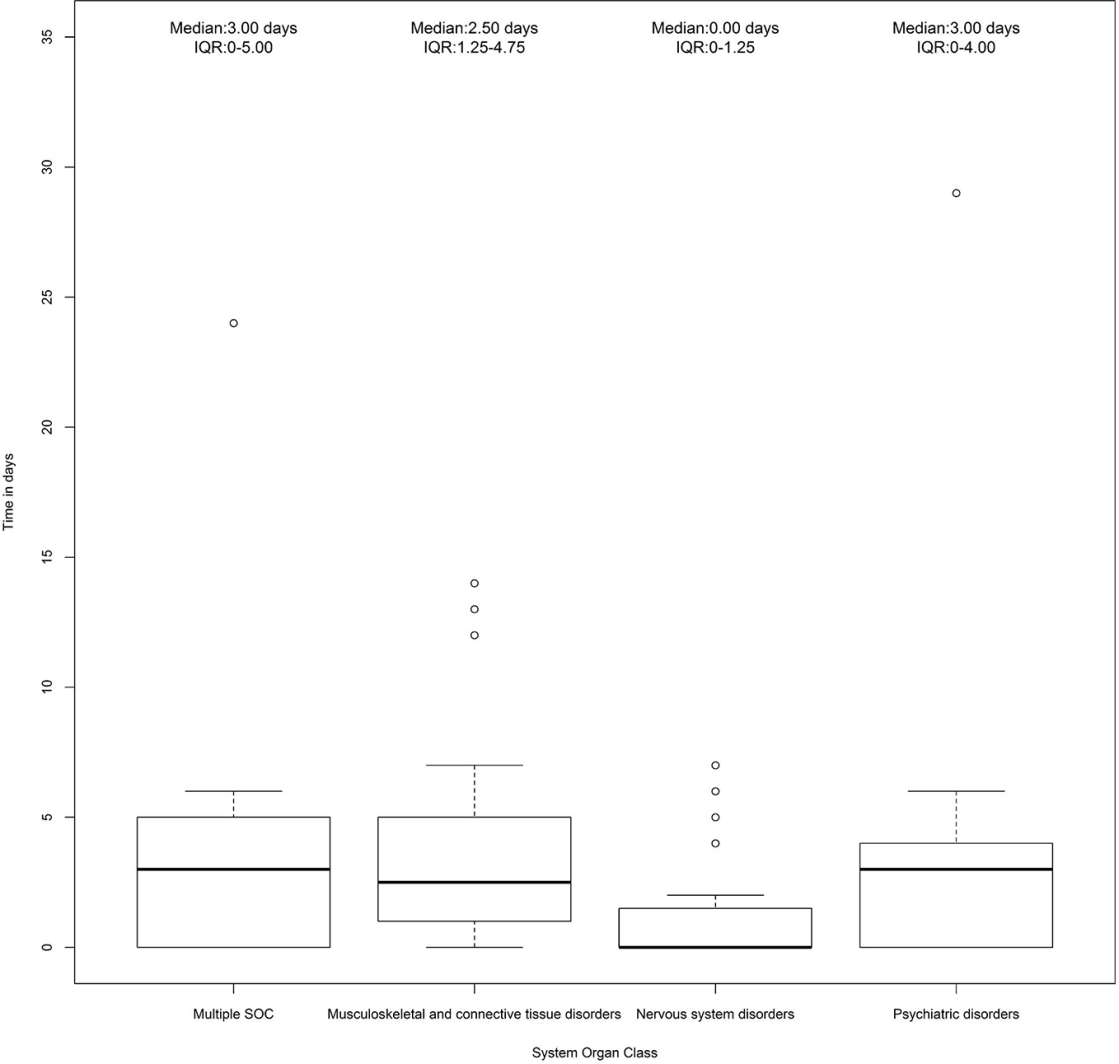
Time to event of cases reporting adverse drug reactions (ADRs) that belong to the System Organ Class (SOC) “Nervous system disorders,” “Psychiatric disorders,” and “Musculoskeletal and connective tissue disorders.” Individual Case Safety Reports (ICSRs) were screened among those reported in the Campania spontaneous reporting system from January 2001 to April 2019.

A total of 87 cases (19.7%) out of 442 ICSRs reported at least one musculoskeletal, neurological, and psychiatric adverse event. Specifically, 46 ICSRs reported a third-generation quinolone as a suspected drug, 34 ICSRs reported a second-generation quinolone, five ICSRs reported a fourth-generation quinolone, and two ICSRs reported a first-generation quinolone. Characteristics of cases associated with musculoskeletal, neurological, and psychiatric adverse events were presented in **Table 3**. Because in each ICSR more than one ADR could be reported, we observed a total of 229 ADRs (2.6 suspected ADRs per ICSR). A total of 132/229 (57.6%) ADRs affected muscles, joints, and the nervous system. Specifically, 63 (47.7%) ADRs belong to the SOC “Nervous system disorders,” 45 (34.1%) to the SOC “Musculoskeletal and connective tissue disorders,” and 24 (18.2%) to the SOC “Psychiatric disorders.” The three most reported ADRs were tremor (16/63; 25.4%), paresthesia (7/63; 11.1%), and headache (7/63; 11.1%) for the SOC “Nervous system disorders”; myalgia (9/45; 20.0%), arthralgia (9/45; 20.0%), and tendinitis (8/45; 17.8%) for the SOC “Musculoskeletal and connective tissue disorders”; insomnia (3/24; 12.5%), hallucination (3/24; 12.5%), and confusion (2/24; 8.3%) for the SOC “Psychiatric disorders.” All musculoskeletal, neurological, and psychiatric adverse events are listed in **Table 4**. The median times to event was 3 days (interquartile range [IQR]: 0–4) for the SOC “Psychiatric disorders,” 0 day (IQR: 0–1.25) for the SOC “Nervous system disorders,” 2.5 (IQR: 1.25–4.75) for the SOC “Musculoskeletal and connective tissue disorders,” and 3 days (IQR: 0–5) for “Multiple SOC” (**Figure 1**).

**Table 4:** Number of adverse drug reactions (ADRs) belonging to the System Organ Class (SOC) “Nervous system disorders,” “Psychiatric disorders,” and “Musculoskeletal and connective tissue disorders” and involving quinolones as suspected drugs recognized in the Campania spontaneous reporting system.

**SOC - Reported Adverse Events** *Preferred term*	**Number** **(%)**
***Nervous system disorders*** *Tremor* *Paraesthesia* *Headache* *Syncope* *Pre-syncope* *Formication* *Dizziness* *Ageusia* *Memory loss* *Anosmia* *Epilepsy* *Altered state of consciousness* *Metallic taste* *Paralysis* *Peripheral neuropathy* *Decreased level of consciousness* *Sudden fall* *Hypotonia* *Afasia* *Drowsiness* *Akathisia* *Burning sensation* *Cerebral Ischemia* *Balance disorder* *Hemiparesis*	**63 (47.7)** 16 (25.4) 7 (11.1) 7 (11.1) 6 (9.5) 5 (7.9) 2 (3.2) 2 (3.2) 1 (1.6) 1 (1.6) 1 (1.6) 1 (1.6) 1 (1.6) 1 (1.6) 1 (1.6) 1 (1.6) 1 (1.6) 1 (1.6) 1 (1.6) 1 (1.6) 1 (1.6) 1 (1.6) 1 (1.6) 1 (1.6) 1 (1.6) 1 (1.6)
**Musculoskeletal and connective tissue disorders** *Myalgia* *Arthralgia* *Tendinitis* *Limb pain* *Tendon pain* *Musculoskeletal pain* *Rhabdomyolysis* *Arthromyalgia* *Hypercreatininemia* *Muscle rigidity* *Muscle tension* *Muscle inflammation* *Muscle weakness*	**45 (34.1)** 9 (20.0) 9 (20.0) 8 (17.8) 5 (11.1) 4 (8.9) 3 (6.7) 1 (2.2) 1 (2.2) 1 (2.2) 1 (2.2) 1 (2.2) 1 (2.2) 1 (2.2)
**Psychiatric disorders** *Insomnia* *Hallucination* *Confusional state* *Agitation* *Temporospatial disorientation* *Restlessness* *Irritability* *Acute psychosis* *Anxiety* *Drug abuse* *Dyssomnia* *Mood swing* *Auditory hallucination* *Mental disorder* *Psychotic disorder* *Panic attack*	**24 (18.2)** 3 (12.5) 3 (12.5) 2 (8.3) 2 (8.3) 2 (8.3) 2 (8.3) 1 (4.2) 1 (4.2) 1 (4.2) 1 (4.2) 1 (4.2) 1 (4.2) 1 (4.2) 1 (4.2) 1 (4.2) 1 (4.2)
***Total***	**132 (100.0)**

#### Risk Factors for Musculoskeletal, Neurological, and Psychiatric Adverse Events

**Figure 2:**
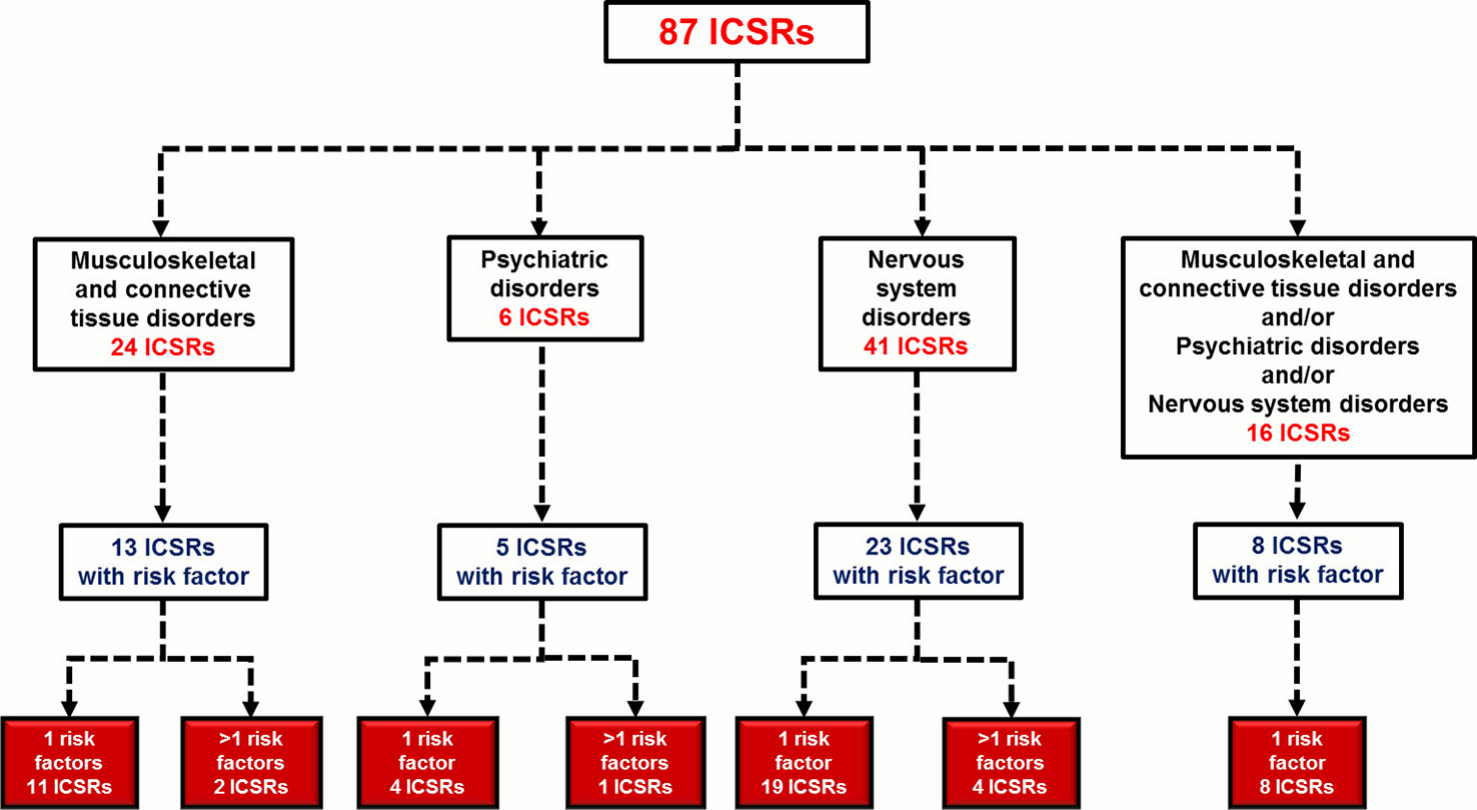
Distribution of Individual Case Safety Reports (ICSRs) with risk factors for the System Organ Class (SOC) “Nervous system disorders,” “Psychiatric disorders,” and/or “Musculoskeletal and connective tissue disorders.” ICSRs were screened among those reported in the Campania spontaneous reporting system from January 2001 to April 2019.

In total, 49 (56.3%) out of 87 ICSRs reported at least one risk factor for the onset of musculoskeletal, neurological, and psychiatric adverse events; seven ICSRs (14.3%) reported more than one risk factor (four ICSRs reported two risk factors and three ICSRs reported three risk factors). The distribution of ICSRs with risk factors for each SOC is reported in **Figure 2**. A total of 59 risk factors were described among cases. The most-reported risk factor was “age greater than 60 years” (41; 69.5%), followed by “therapeutic indication” (10; 16.9%), “renal failure” (3; 5.1%), “organ transplantation” (2; 3.4%), “use of corticosteroid” (2; 3.4%), and “history of side effects” (1; 1.7%) (**Table 5**). Potentially wrong therapeutic indications were tonsillitis, ovarian cysts, fever, cataract, tracheitis, flu, tooth pain, renal colic, drug abuse, or self-harm. For cases with two risk factors “age” with “renal failure” or “therapeutic indication” or “history of side effect” or “use of a corticosteroid” were simultaneously reported. For cases with three risk factors were simultaneously reported: “age”/”renal failure”/”organ transplant” in two ICSRs; “age”/”use of corticosteroid”/”therapeutic indication” in one ICSR. All cases of organ transplantation were related to heart transplants.

**Table 5:** Number of risk factors reported in Individual Case Safety Reports (ICSRs) with adverse drug reactions (ADRs) belonging to the System Organ Class (SOC) “Nervous system disorders,” “Psychiatric disorders,” and “Musculoskeletal and connective tissue disorders.” ICSRs were screened among those reported in the Campania spontaneous reporting system from January 2001 to April 2019.

	Age (60 years)	Renal failure	Organ transplantation	Use of corticosteroid	History of side effects	Therapeutic indication
**Musculoskeletal and connective tissue disorders** (14 ICSRs)	12	1	1	1	–	1
**Psychiatric disorders** (5 ICSRs)	4	1	–	–	–	1
**Nervous system disorders** (23 ICSRs)	20	1	1	1	1	5
**Musculoskeletal and connective tissue disorders and/or** **Psychiatric disorders and/or Nervous system disorders** (16 ICSRs)	5	–	–	–	–	3
**Total**	41	3	2	2	1	10

## Discussion

### ICSRs Involving Quinolones in the Italian Spontaneous Reporting System

From January 2002 to March 2019, 12,059 ICSRs, covering 21,941 ADRs, reporting a quinolone as a suspected drug, were sent to the Italian spontaneous reporting system. Quinolones-induced ADRs occurred more commonly in the age groups 18–65 years and >66 years and in a slightly higher percentage of female patients (51%), even though almost 8% of cases missed gender information Quinolones that were more frequently reported as suspected (> 80% of all ICSRs) were ciprofloxacin and levofloxacin. Out of 21,941 ADRs, almost 20% (n = 4,418) referred to the SOC “Nervous system disorders,” “Musculoskeletal and connective tissue disorders,” or “Psychiatric disorders.” A higher number of musculoskeletal and neurological ADRs were found compared to psychiatric ones (1,681 ADRs belonging to the SOC “Musculoskeletal and connective tissue disorders” and 1,619 ADRs belonging to the SOC “Nervous system disorders” vs. 1,118 ADRs belonging to the SOC “Psychiatric disorders”) and some differences were noticed among quinolone generations. For instance, looking at the number of ADRs, we found that the number of ciprofloxacin-induced musculoskeletal and neurological ADRs was similar (n = 491 and n = 495, respectively) but also higher compared to psychiatric ones (n = 265), while the number of levofloxacin-induced musculoskeletal ADRs was higher compared to both neurological and psychiatric ones (1,025 musculoskeletal ADRs vs. 796 neurological ADRs and 682 psychiatric ADRs). ROR showed that second-generation quinolones were associated with a lower reporting probability of musculoskeletal, neurological, and psychiatric ADRs compared to the third generation of quinolones. Moreover, fourth-generation quinolones were associated with a higher reporting probability of musculoskeletal ADRs compared to second- or third-generation quinolones but not of psychiatric ADRs if compared to third-generation quinolones. First-generation quinolones were instead associated with a higher reporting probability of neurological ADRs compared to quinolones of the second or third generation.

In our opinion, the higher number of ICSRs related to levofloxacin and ciprofloxacin could be related to the higher utilization of those medicines in Italy. Indeed, levofloxacin and ciprofloxacin appear both on the list of the most used antibiotics (data related to prescriptions reimbursed by the Italian Health System in 2018) in Italy together with amoxicillin and clavulanic acid, ceftriaxone, cefixime, and fluconazole (Italian Medicine Agency, 2017). The well-established use of levofloxacin and ciprofloxacin is not a prerogative of the Italian pharmaceutical market rather it is extended to other countries, including the US where both drugs dominated the antibiotic pharmaceutical market for almost 20 years (Almalki et al., 2017). From the same observation, it is possible to deduce that the lower number of ICSRs related to first-generation quinolones could be related to their lower use in the clinical practice. Indeed, the increasing marketing authorization of newer quinolone generations with an increased antimicrobial spectrum of activity, improved pharmacokinetic characteristics and lower cost (Patel and Goldman, 2016) resulted in a lesser utilization of older ones, such as cinoxacin, pipemidic acid, and norfloxacin.

Literature data are quite poor with regard to the direct comparison between quinolones generations in terms of musculoskeletal, neurological, and psychiatric ADRs. Indeed, to our knowledge, the great many of the available studies have evaluated safety differences between every single quinolone. For instance, a study performed by Leone et al., using data from the Italian spontaneous reporting system in the Regions of Emilia Romagna, Lombardia, and Veneto, showed that among third- and second-generation quinolones, levofloxacin was associated with the highest rate of serious tendinitis, while no reports related to tendon disorders were reported for ofloxacin, rufloxacin, pefloxacin, and moxifloxacin. Levofloxacin was also associated, together with rufloxacin, with the highest number of psychiatric ADRs (Leone et al., 2003). On the other hand, a survey carried out by the World Health Organization (WHO) among data from the Australian ADRs Advisory Committee revealed that ciprofloxacin, a second-generation quinolone, was the most common causal agent of the Achilles tendonitis or rupture (World Health Organization, 2003), while another study showed that levofloxacin ranked first for tendon ruptures (Melhus, 2005). Lastly, Zhanel et al. carried out a literature review highlighting that neurological and psychiatric ADRs, mainly dizziness and headache, have been reported for many of the new quinolones and that trovafloxacin causes the highest incidence of dizziness while levofloxacin and sparfloxacin have not been observed to produce this ADR. The incidence of headache was similar for all quinolones, with roughly 1% to 5% of patients treated reporting this ADR (Zhanel et al., 1999). Taken into account the few and sometimes conflicting data available on these specific associations, what we have found might be explained by differences in the chemical structure and/or pharmacokinetic properties of quinolone generations. Although the exact pathologic mechanisms underlying quinolone-induced musculoskeletal ADRs are still poorly understood, it was suggested that such antibiotics might alter tendon fibroblast metabolism, leading to collagen damage (Shakibaei et al., 2000; Williams et al., 2000). These ADRs seem to be rare, affecting <1 in 5,000 patients receiving quinolones with an overall incidence of three cases per 1,000 patient years, even though some quinolones, such as pefloxacin, show an increased risk (Ball, 2003). On the other hand, the occurrence of both neurological and psychiatric ADRs may have several underlying factors, including the higher penetration of quinolones across the blood-brain barrier (such as pefloxacin that shows convulsant activity) (Kushner et al., 2001; Sarro and Sarro, 2012) and the interaction with the activity of neurotransmitters, including the displacement of GABA from its receptors GABA-A or the direct effect on excitatory brain pathways on N-methyl-D-aspartate and α-amino-3-hydroxy-5-methyl-4-isoxazolepropionic acid receptors (Mehlhorn and Brown, 2007; Rubio-Nazabal et al., 2013). Differences in binding potency of quinolones to the GABA-A receptors may explain the variability in neurotoxic effects (Akahane et al., 1989). Lastly, differences in the chemical structure of quinolones may further contribute to an increase in the risk of neurological and psychiatric ADRs. For instance, levofloxacin, which is the left optical isomer of ofloxacin, is associated with fewer neurological and psychiatric ADRs when compared with ofloxacin and the reason seems to be the influence of the R(+) isomer of ofloxacin that could contribute to the ADR profile of levofloxacin (Tomé and Filipe, 2011). On the other hand, Schwartz MT et al. reported that even though ofloxacin has an increased permeability in the central nervous system, fewer cases of neurotoxicity have been reported for ofloxacin compared to ciprofloxacin (Schwartz and Calvert, 1990).

### ICSRs Involving Quinolones in the Campania Region

Starting from 2001 until April 2019, 442 ICSRs reporting at least one quinolone as suspected were sent to the Campania Pharmacovigilance Regional Center. In line with national data, quinolones-induced ADRs occurred in patients with a median age of 55 (IQR 36–70) and in a slightly higher percentage of female patients compared to male ones. Almost 65% of ICSRs reported ADRs that were classified as not serious and almost 80% of ICSRs reported ADRs that had a positive outcome (recovered or improvement). In line with the national data, quinolones most commonly reported as suspected were levofloxacin and ciprofloxacin. We found that more than 80% of all ICSRs came from healthcare professionals. Eighty-seven ICSRs, covering 229 ADRs, reported at least one musculoskeletal, neurological or psychiatric ADR. All ADRs have presented within few days from the beginning of the antibiotic therapy. As for any other SOC, quinolones most commonly involved as suspected were third-generation ones, followed by second-generation agents. Lastly, we have found that 49 ICSRs reported at least one risk factor for the onset of musculoskeletal, neurological, and psychiatric ADRs and, among these, eight ICSRs reported more than one risk factor. The most commonly reported risk factors were “age greater than 60 years” (n = 41) and “therapeutic indication” (n = 10).

It is not surprising that quinolones-induced ADRs occurred more commonly in adult patients, considering that, apart from ciprofloxacin that is indicated in children for the treatment of inhalation anthrax, complicated urinary tract infections, and pyelonephritis, these antibiotics are contraindicated in the pediatric age (Italian Medicine Agency, 2016; Patel and Goldman, 2016) and therefore their use is mainly expected in adults.

With regard to the gender distribution, in our opinion, the higher percentage of female patients experiencing quinolones-induced ADRs could be attributed to gender differences in the pharmacokinetic and pharmacodynamic behavior of any drug; as matter of fact, the risk of ADR for women of developing an ADR is 1.5- to 1.7-fold higher compared to men (Rademaker, 2001; Zopf et al., 2008). This was previously reported for other drug classes-induced ADRs in other studies (Scavone et al., 2018b; Mascolo et al., 2019). Furthermore, Tomé AM et al. found a higher prevalence of quinolones-induced ADRs in female patients (Tomé and Filipe, 2011).

According to our results, previous studies based on the spontaneous reporting system show that quinolones-induced ADRs are mainly not serious (Lapi et al., 2010; Oreagba et al., 2017). Furthermore, literature data suggested that quinolones-induced musculoskeletal ADRs usually resolve within a few weeks; however, in some cases, they may persist and require surgical intervention (Williams et al., 2000). Similarly, few case reports showed that, in case of ADRs affecting the central nervous system, after the discontinuation of the suspected drug a complete resolution of patient's symptoms is frequently observed (Kiangkitiwan et al., 2008; Slobodin et al., 2009; Mazhar et al., 2016; Higdon et al., 2017).

As explained for the national data, also data on drug utilization in the Campania Region showed that quinolones were among antibiotics most commonly prescribed in 2017 together with penicillins and cephalosporins (OsMed, 2017). Indeed, the Campania Region stands out for the higher utilization of antibiotics, including quinolones, compared to the rest of the Italian Regions (Sarnelli et al., 2017).

In our opinion, the type of ADR that we have analyzed could explain the higher number of reports from healthcare professionals. Indeed, musculoskeletal, neurological and psychiatric ADRs require a diagnosis that is based on a good history and examination made by a physician. For instance, for the diagnosis of tendinopathy, ultrasound and magnetic resonance imaging is helpful in identifying tendon tear or rupture (Ng and Naughton, 2007). Nevertheless, few cases were reported by the patient/citizen; as previously reported (Scavone et al., 2018a), in recent years a positive trend for reports from patients is affecting the spontaneous reporting system on the entire Italian territory.

With regard to the most common reported PTs, in line with our results, literature data suggested that among neurological and psychiatric SOCs the most reported ADRs are a headache, dizziness, confusion, impaired thinking, insomnia and psychosis (Mandell and Tillotson, 2002). Similarly, musculoskeletal ADRs are usually represented by localized pain, swelling, tenderness, erythema, or itchiness over tendon sites (Van Der Linden et al., 2001; Khaliq and Zhanel, 2003). The median time to event that we have found is in line with the latency period reported by other studies (from few hours to few weeks) (Popescu, 2018; Van Der Linden et al., 2001; Ng and Naughton, 2007; Akali and Niranjan, 2008; Golomb et al., 2015) as well as with the suggested mechanism and factors underlying the occurrence of ADRs that we have evaluated (Akahane et al., 1989; Schwartz and Calvert, 1990; Lipsky and Baker, 1999; Kushner et al., 2001; Tomé and Filipe, 2011; Rubio-Nazabal et al., 2013).

In line with our findings, Ball P reported that the most common predisposing factor for quinolones-induced ADRs is advanced age (risk increases with age after 60 years) (Ball, 2003). In this regard, one can suppose that the altered renal function, which is relatively common in older people, may affect the excretion of quinolones leading to higher risk in the occurrence of musculoskeletal, neurological, and psychiatric ADRs. Furthermore, literature data suggest that neurological and psychiatric ADRs are of particular concern in the elderly population since many of signs and symptoms of central nervous system disorders, including confusion, weakness, tremor, are often erroneously attributed to old age remaining unreported (Chow et al., 2004; Stahlmann and Lode, 2010). Lastly, other conditions and factors, including infections, pharmacotherapy, cardiovascular diseases, metabolic conditions, and trauma, increase the risk of psychiatric ADRs in the geriatric population (Mattappalil and Mergenhagen, 2014). In line with these literature data as well as with our findings, the results of a case-crossover study, based on data from the Health Improvement Network database (almost 6.4 million patients from general practices in UK), showed that the use of quinolones is strongly associated with an increased risk of Achilles tendonitis (OR=4.3, 95% CI: 3.2–5.7) and tendon rupture (OR=2.0, 95% CI: 1.2–3.3) and that this association is stronger among patients older than 60 years (OR 8.3 vs. 1.6) (Wise et al., 2012). In our study, the risk factor “therapeutic indication” mainly consisted of the prescription of quinolones for the treatment of not authorized indications, including tonsillitis, ovarian cysts, fever, cataract, tracheitis, flu, tooth pain, and renal colic. Indeed, as reported in guidelines related to the management of respiratory and urinary tract infections, quinolones are not recommended for the treatment of infections that we have found among our cases (European Association of Urology, 2018). The overprescription of quinolones or their use not in accordance with the Summary of Product Characteristics is a common phenomenon in primary care and it is one of the leading cause of unnecessarily exposes patients to side effects as well as antimicrobial resistance (Dekker et al., 2015; Oreagba et al., 2017). According to Belliveau PP et al., almost 46% of levofloxacin prescriptions were replaceable by other conventional antimicrobial therapies (Belliveau et al., 2000). Literature data suggest that two main reasons may underline the higher prescription of quinolones, including the fact that prescribers intend to use a broader-spectrum agent than necessary and, second, since patients expect to be prescribed medication at the end of the visit, clinicians tend to increase their antibacterial-prescribing habit to ensure the patients are satisfied with their care (Almalki et al., 2017).

Furthermore, the inappropriate use of quinolones has a significant influence on drug resistance as assessed from the last report of surveillance of antimicrobial resistance in Europe (European Centre for Disease Prevention and Control, 2018). Considering this widespread phenomenon, the Campania Pharmacovigilance Regional Centre has promoted this study as part of initiatives to ensure drug safety and to improve appropriate drug use during the last years (Sessa et al., 2016b; Sessa et al., 2016a; Scavone et al., 2017). Lastly, even though we have found only two cases with “use of corticosteroid” as risk factor, the risk of the concomitant exposure to quinolones and systemic or local corticosteroids should not be underestimated. Indeed, the interaction underlying this association is one of the most common antecedent of Achilles tendon rupture (Ford and Debender, 1979; Kleinman and Gross, 1983; Newnham et al., 1991).

## Strengths and Limitation

This study has several limitations and strengths. Our study was based on data from the spontaneous reporting system, which is affected by several limitations that mainly include the underreporting phenomenon, the inaccurate and incomplete information, and the lack of clinical data. Considering these intrinsic limitations, we cannot rule out the presence of information that were not listed in ICSRs, such as those related to specific risk factors (patients' age, concomitant diseases and medications, history of previous side effects to quinolones, therapeutic indications of quinolones) that may have contributed to the occurrence of ADRs. Therefore, we may have underestimated the number of cases with risk factors for musculoskeletal, neurological, and psychiatric ADRs. In addition, considering the main features of the RAM system that imply the inability of performing a case by case assessment, we were not able to perform an adjusted analysis for confounding factors, such as comorbidities, concomitant medications, patients' age, and unapproved therapeutic indications. Lastly, the description of risk factors was performed on a small number of regional ICSRs, therefore, our results need to be interpreted with caution.

Notwithstanding these limitations, our study has many strong points. The first, in our opinion, is the time that was chosen for this analysis. Indeed, we have carried out our safety analysis immediately after the safety review of the EMA on quinolone antibiotics to provide new and up-to-date data on quinolones-induced musculoskeletal, neurological, and psychiatric ADRs from the Italian clinical practice. Second, we have analyzed a huge amount of national data providing statistical comparison, ROR, its 95% CI and the Chi-square test, between quinolone generations and a lower/higher probability of reporting musculoskeletal, neurological, and psychiatric ADRs. Third, we have provided a descriptive analysis of regional safety data to describe the most common reported risk factors that may have contributed to the occurrence of ADRs that we have analyzed. Lastly, it should be highlighted that data obtained from the spontaneous reporting system represent a valuable source of medical data.

## Conclusion

The use of quinolones has undoubtedly advanced the treatment of several infections. However, in recent times new safety concerns related to the risk of quinolones-induced musculoskeletal, neurological and psychiatric ADRs have emerged. To prevent the occurrence of these serious, disabling and potentially permanent side effects in patients treated with quinolone antibiotics, the EMA decided for the suspension of the marketing authorization of cinoxacin, flumequine, nalidixic acid, and pipemidic acid and the restriction in the utilization of the remaining quinolones. The EMA decision is supported from the views of patients, healthcare professionals and academics presented at EMA's public hearing. Besides, literature data confirm that the inappropriate use of quinolones is a common phenomenon that frequently predisposes patients to the occurrence of ADRs.

Starting from these concerns and considering the recent recommendations endorsed by the EMA, we decided to carry out a study using data from the Italian spontaneous reporting system to evaluate cases of musculoskeletal, neurological, and psychiatric ADRs that reported a quinolone as suspected drug. In our study, we have found that from January 2002 to March 2019, 12,059 ICSRs sent to the Italian spontaneous reporting system reported a quinolone as suspected drug, mainly levofloxacin, ciprofloxacin and moxifloxacin. Out of 12,059 ICSR, almost 20% were related to the SOC “Nervous system disorders,” “Musculoskeletal and connective tissue disorders,” or “Psychiatric disorders.” We have found that third-generation quinolones were always associated with a higher reporting probability of musculoskeletal, neurological, and psychiatric ADRs compared to the second generation of quinolones. From January 2001 to April 2019, 442 ICSRs sent to the Campania spontaneous reporting system reported a quinolone as suspected drug. Of these ICSRs, 87 reported at least one musculoskeletal, neurological, and psychiatric ADR. The most commonly reported ADRs were tremor, paresthesia, and headache for the SOC “Nervous system disorders”; myalgia, arthralgia, and tendinitis for the SOC “Musculoskeletal and connective tissue disorders”; insomnia, hallucination, and confusion for the SOC “Psychiatric disorders.” Risk factors were described in 49 ICSRs; these were mainly represented by “age greater than 60 years” and “therapeutic indication.”

Based on the results of our study, especially those related to the different reporting probability of musculoskeletal, neurological, and psychiatric ADRs among quinolone generations, we believe that a better understanding of the structure-related side effects of quinolones may improve the predictability of such ADR. Furthermore, since the inappropriate prescription of quinolones is one of the leading causes of ADRs, a rational prescribing from physicians can provide the best outcome in terms of clinical care as well as lower risk of ADRs and antibiotic resistance. In this context, it is widely accepted that pharmacovigilance activities play a key role in contributing to the protection of public health since they are aimed at analyzing, evaluating, understanding, and preventing ADRs and any critical issues related to the use of medicines, to guarantee a favorable benefit/risk ratio for the population (World Health Organization, 2002; European Medicine Agency, 2017). For all these reasons, some Italian deputies have recently proposed to include pharmacovigilance activities within the so-called “essential levels of care” (Livelli Essenziali di Assistenza, LEAs) that define the standards of health services provided by the Italian health system (Italian Senate, 2019).

## Data Availability Statement

The datasets generated for this study will not be made publicly available. The distribution of pharmacovigilance data needs the authorization from the Italian Medicine Agency except for national aggregated data available at https://www.aifa.gov.it/sistema-ram.

## Ethics Statement

Ethical review and approval were not required for the study on human participants in accordance with the local legislation and institutional requirements. Written informed consent from the participants' legal guardian/next of kin was not required to participate in this study in accordance with the national legislation and the institutional requirements.

## Author Contributions

Drafting the work and revising it for important intellectual content: AM, CS, RR, LS, CR, LB, and AC. Substantial contributions to the acquisition, analysis, or interpretation of data for the work: AM and CS. Final approval of the version to be published: AM, CS, RR, LS, CR, LB, and AC. Agreement to be accountable for all aspects of the work in ensuring that questions related to the accuracy or integrity of any part of the work are appropriately investigated and resolved: AM, CS, RR, LS, CR, LB, and AC. Developed the concept and designed the study: LB and AC. Wrote the paper: AM and CS. 

## Conflict of Interest

The authors declare that the research was conducted in the absence of any commercial or financial relationships that could be construed as a potential conflict of interest.
